# A Large Isolated Congenital Left Circumflex Artery-to-Right Atrial Fistula in a 9-Year-Old Child

**DOI:** 10.3389/fped.2020.00051

**Published:** 2020-02-20

**Authors:** Xin Li, Jun An, Shuai Wang, Wanli Lu, Zhigang Liu, Yili Wu, Fengjuan Jiao

**Affiliations:** ^1^Department of Pediatrics, TEDA International Cardiovascular Hospital, TEDA, Tianjin, China; ^2^Department of Pediatric Cardiac Surgery, TEDA International Cardiovascular Hospital, TEDA, Tianjin, China; ^3^Department of Biochemistry and Molecular Biology, College of Life Sciences, Nankai University, Tianjin, China; ^4^Shandong Collaborative Innovation Center for Diagnosis, Treatment and Behavioral Interventions of Mental Disorders, Institute of Mental Health, Jining Medical University, Jining, China; ^5^Shandong Key Laboratory of Behavioral Medicine, School of Mental Health, Jining Medical University, Jining, China; ^6^Department of Great Blood Vessel and Cardiac Surgery, TEDA International Cardiovascular Hospital, TEDA, Tianjin, China; ^7^Collaborative Innovation Center for Birth Defect Research and Transformation of Shandong Province, Jining Medical University, Jining, China

**Keywords:** coronary artery fistula, congenital, heart defects, pediatrics, surgical procedures

## Abstract

Isolated congenital coronary artery fistula (ICCAF) is an exceedingly rare anomaly in which there is a direct abnormal connection between a coronary artery and other cardiac chambers or any of great vessels. The left circumflex artery (LCX) is the least common source of ICCAF. Here we reported a rare case of large ICCAF originated from the LCX in a 9-year-old boy. He presented fatigability, murmurs and NYHA class II. Echocardiography and cardiac CT revealed that an aneurysmal dilatation of the LCX along with the dilated coronary sinus entered into the right atrium (RA) through the great cardiac vein. However, it showed that the dilated LCX directly drained into the RA by coronary angiography, which was confirmed by the surgery. During the surgical procedure, the LCX fistula was identified in a 3^*^3 cm bulbous structure, the aneurysmal dilation of RA tissue. The end of fistula was located in the lower-middle interatrial septum, which was near the coronary sinus and above the opening of inferior vena cava (IVC). Transcardiac chamber closure with cardiopulmonary bypass (CPB) was successfully performed for the correction of the fistula. It indicated that preoperative angiography is essential to define the details of large ICCAF with aneurysmal dilation. Moreover, transcardiac chamber closure with CPB is the optimal procedure for the treatment of large ICCAF, while interventional catheterization is not feasible due to the presence of aneurysmal dilation of the LCX. The description of this rare case might have great value for the diagnosis and treatment of large ICCAF originated from the LCX.

## Background

Isolated congenital coronary artery fistula (ICCAF) is an exceedingly rare anomaly in which there is a direct abnormal connection between a coronary artery and one of the four cardiac chambers or any of great vessels without other abnormal cardiac structure (1–3). The incidence of ICCAF is 0.002% in the general population and ICCAF accounts for 0.4% in patients with cardiac malformations (4–6). The left circumflex artery (LCX) fistula is the most rare type of ICCAF (7, 8). Here we reported a rare case of large ICCAF originated from the LCX in a 9-year-old boy.

## Case Presentation

**Figure 1:**
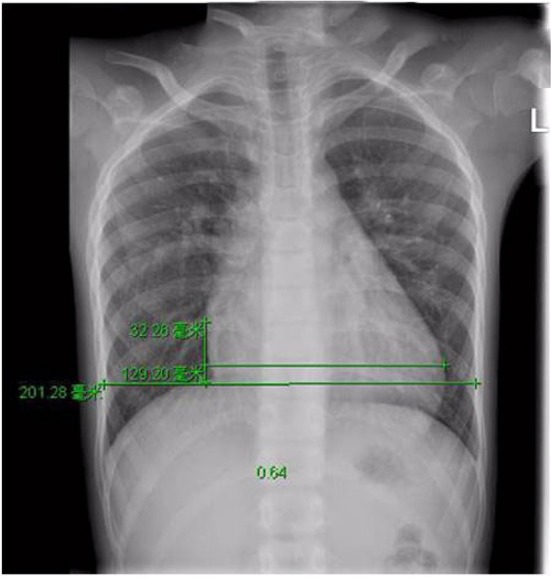
Pre-operative Chest X-ray film view showed increased pulmonary flow and cardiomegaly, with a cardiothoracic area ratio of 0.64.

**Figure 2:**
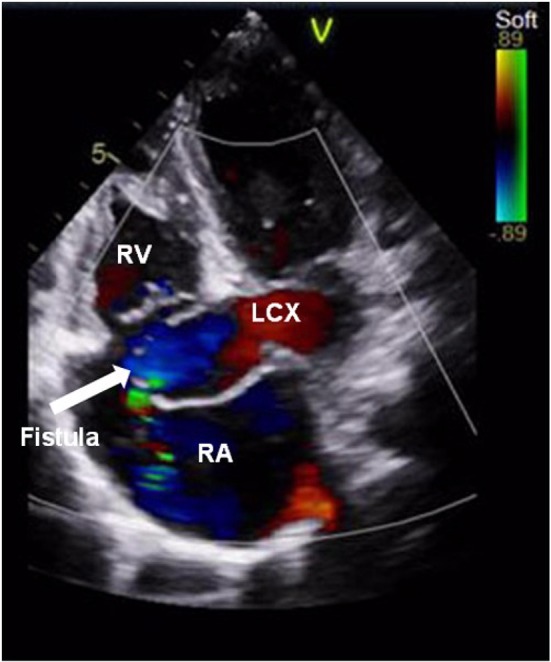
Color Doppler examination showed a coronary artery fistula from the left circumflex artery to the right atrium. The arrow points to the fistula. RV, right ventricle; LCX, left circumflex artery; RA, right atrium.

**Figure 3:**
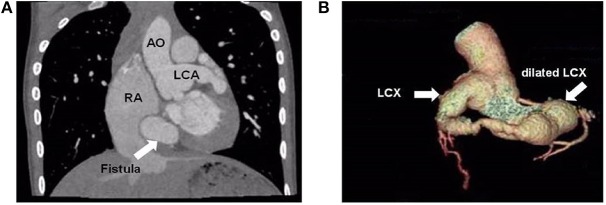
Cardiac computed tomographic view the dilated left main coronary artery and circumflex artery. **(A)** Cardiac computed controlled X-ray shows dilated left main coronary artery and right atrium. The arrow points to the fistula. **(B)** Three-dimensional reconstructed computed tomographic demonstrates the dilated left circumflex artery. The distal left circumflex artery showed an aneurysmal dilatation. AO, aorta; LCA, left coronary artery; RA, right atrium; LCX, left circumflex artery.

**Figure 4:**
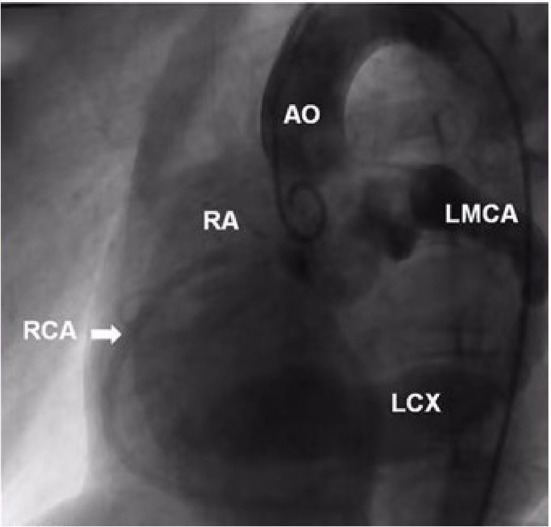
Ascending aorta and coronary angiography view showed the dilated left main and circumflex coronary arteries. The distal LCX showed a stage of aneurysmal dilatation. AO, aorta; RA, right atrium; RCA, right coronary artery; LMCA, left main coronary artery; LCX, left circumflex artery.

A 9-year-old boy (weight 29.55 kg and height 139 cm) entered the hospital because of easy fatigability in the past 3 years. Continuous blowing murmur was found in the precordial region, and NYHA class II was defined. On auscultation, a grade 2/6 continuous blowing murmur could be heard at the third and fourth right intercostal spaces close to the sternum. The chest film demonstrated increased peripheral pulmonary vascularity and moderate to marked cardiomegaly, especially the right heart, with a cardiothoracic ratio of 0.64 (Figure 1). The electrocardiogram result showed sinus arrhythmia. In echocardiography and contrast-enhanced computed tomography (cardiac CT), the left main and circumflex coronary arteries showed an aneurysmal dilatation, which joined with the dilated coronary sinus (15 mm) through the great cardiac vein, and then entered into the right atrium (RA) (Figures 2, 3). Subsequent coronary angiography revealed the distal LCX with an aneurysmal dilatation directly extended into the RA, while it failed to delineate the anatomy of the fistula (Figure 4). The surgery is necessary for this patient to keep healthy. Because the location of the fistula could not be defined by echocardiography and coronary angiography, the surgical operation was necessary to locate the fistula and treat it.

**Figure 5:**
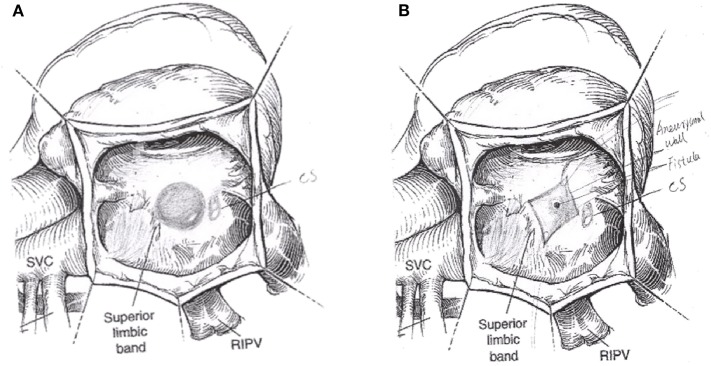
The operation schematic diagram showed the distal aneurysm-like left circumflex artery was exposed within the right atrium **(A)**. The LCX fistula was shown by cutting the aneurysm **(B)**. CS, coronary sinus; SVC, superior vena cava; RIPV, right inferior pulmonary vein.

Median sternotomy and pericardiotomy were performed. External cardiac exploration showed that the end of LCX was a long segment ectasia that was connected to RA. Under cardiopulmonary bypass (CPB), the specific location of the LCX-RA fistula was determined. The CPB was instituted with bicaval drainage and aorta cannulation with mild hypothermia (33–35°C). During parallel circulation process, the RA was opened. A 3^*^3 cm bulbous structure was found. On the side surface of bulbous structure, there was a 4 mm opening that sprayed more blood and the coronary sinus opening compressed by the bulbous structure was located inferiorly and posteriorly. The left heart was vented via the atrial septum. The bulbous structure was opened, and a communication with LCX distal segment was found in the deep, that was the location of the LCX fistula. The end of fistula was located in the lower-middle interatrial septum, which was near the coronary sinus and above the opening of inferior vena cava (IVC). Then we found the component of the bulbous structure was RA tissue with aneurysmal dilation (Figure 5). Several interrupted pledgeted stitches was used to close the fistula and there was no any effluent blood, while the cardioplegia was repeatedly given. The fistula was repaired, and the aneurysmal wall was resected and trimmed subsequently.

**Figure 6:**
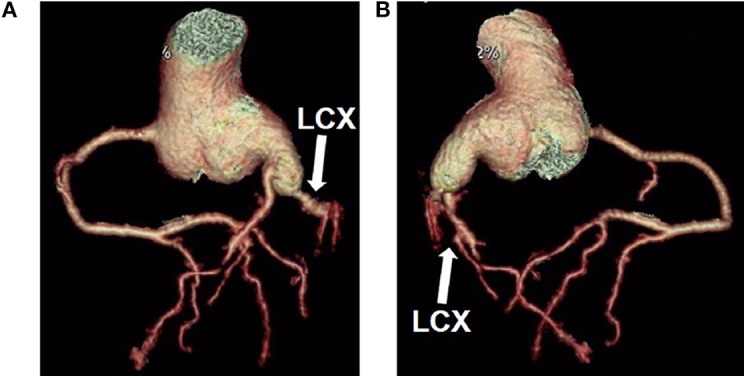
Cardiac computed tomographic showed constriction of the left coronary artery and circumflex artery **(A,B)**. LCX, left circumflex artery.

The whole 157 min procedure involved 35 min cross-clamp and 62 min CPB.120 min ventilation and 18 h ICU stay were applied postoperatively. After weaning from CPB, no electrocardiographic change of myocardial ischemia was detected. The postoperative ECG showed that sinus rhythm was recovered. The postoperative course was uneventful. Oral aspirin was given for 12 months. No thrombus was found at 9 month follow-up, although a thrombus was found at the end of LCX at 1 and 3 months postoperative follow-up, respectively. Twelve months after the operation, cardiac CT showed that the diameter of proximal LCX and left main coronary artery was significantly reduced, and there was no abnormal connection between the LCX and the RA (Figure 6).

## Discussion

Coronary artery fistula (CAF) can be congenital or acquired. Non-isolated congenital CAF was associated with other congenital heart diseases including tetralogy of Fallot or pulmonary atresia, while isolated congenital coronary artery fistula (ICCAF) occurs in structurally normal hearts (9). Patients with ICCAF are usually asymptomatic in pediatric population, and have symptoms or complications more than 20 years old (2). The duration and severity of the clinical symptoms depend on the amount of blood shunt, the size of the fistula and the resistance of the drainage chamber. Clinically, patients with ICCAF may present with palpitations, cyanosis, dyspnea on exertion, symptoms of angina, bacterial endocarditis or heart failure (10). Moreover, continuous murmur with local tremor or systolic and diastolic murmurs at the precardiac area could be heard in most patients with ICCAF (11). In this case, the patient was easy fatigability and presented with a continuous heart murmur in precordium area, and NYHA class II, which may be associated with the large blood flow of the fistula.

The LCX was the least common source of ICCAF, and the right heart chambers were the most common location of drainage (1, 8). Up until now, clinical experience with this rare anomaly have been published in fewer case reports or short series involving a few patients (12). However, most of the reports described the circumflex artery fistulae in patients aged more than 20 years and had clinical symptoms, such as congestive heart failure (13), palpitations and dyspnea (14), atrial fibrillation and angina (15). Hou and colleagues reported 29 LCX fistulas patients in which LCX to the RA fistulas accounted for 41% (12 of 29), whereas they didn't describe the key factors of the cases, e.g., their age, gender, whether they had a large isolated congenital LCX-to-RA fistula, and whether they had obvious clinical symptoms and associated cardiac diseases (16). A large ICCAF draining from the LCX to the RA was found in this pediatric patient, and the end of the ICCAF was located in the lower-middle interatrial septum, which was near the coronary sinus and above the opening of inferior vena cava (IVC).

Echocardiography is an important primary non-invasive tool for identifying the anomalous origin of CAF. In general, echocardiography can show the location and type of the CAF, including the course and drainage site of coronary artery, while it didn't delineate the exact anatomy of the fistula. So far, coronary angiography remains the golden standard imaging tool for diagnosing coronary anomalies and can be used as a diagnostic and therapeutic procedure (17). In this case, echocardiography and cardiac CT showed that the dilated coronary artery was connected with dilated coronary sinus and outlet stenosis of coronary sinus, while coronary angiography showed the ICCAF draining from the distal aneurysm-like LCX to the RA. However, the location of the fistula was not delineated. Liang and Ko (18) reported that a pediatric CAF leading to a dilated coronary artery and secondary aneurysmal formation may be considered as an indication for surgical treatment.

The coronary arteriovenous fistulas are divided into five types according to the chamber or vessel into which they drain: Type I (draining into the right atrium), Type II (draining into the right ventricle), Type III (draining into the pulmonary artery), Type IV (draining into the left atrium), and Type V (draining into the left ventricle) (3). The ICCAF of this patient belongs to Type I coronary arteriovenous fistula, and the ligation of the CAF distal to the origin, a type of surgery without CPB, was recommended for the correction of the fistula. Because it was difficult to make sure the specific location of the LCX-RA fistula, so transcardiac chamber closure with CPB was performed for the correction of the fistula (3). The left main coronary artery and the LCX were dilated obviously, and the end of LCX enters into the RA with aneurysmal dilatation. The LCX fistula was identified in the 3^*^3 cm bulbous structure, the aneurysmal dilation of RA tissue. The end of fistula was located in the lower-middle interatrial septum, which was near the coronary sinus and above the opening of inferior vena cava (IVC). The coronary sinus opening was compressed by the bulbous structure, which may be the main reason for the abnormal dilation of the coronary sinus. Transcardiac chamber closure with CPB was successfully performed without any complications listed in the studies that Hou and colleagues had been reported (16).

## Conclusions

Herein, we present a pediatric case with a large ICCAF stemmed from the aneurysmal LCX and draining into the RA. Due to the special anatomical structure of the ICCAF in this case, the position of the fistula was not accurately defined by echocardiography, cardiac CT and coronary angiography. Based on the clinical presentation and special cardiac pathology, individualized therapeutic strategy was chosen. Transcardiac chamber closure with CPB was successfully performed without any complication. The description of this rare case might have great value for the diagnosis and treatment of large ICCAF originated from the LCX.

## Data Availability Statement

All datasets generated for this study are included in the article/supplementary material.

## Ethics Statement

This study was carried out in accordance with the recommendations of clinical practice guidelines of China (Chinese Medical Association) with written informed consent from all subjects. All subjects gave written informed consent in accordance with the Declaration of Helsinki. The protocol was approved by the Ethical Committee of TEDA International Cardiovascular Hospital.

## Author Contributions

XL, JA, and SW collected and analyzed the data. XL and FJ wrote the manuscript. XL, YW, and FJ revised the manuscript. XL and WL managed the patient. WL and ZL participated in the surgery. WL provided a photo of the operation and answers the operation questions.

### Conflict of Interest

The authors declare that the research was conducted in the absence of any commercial or financial relationships that could be construed as a potential conflict of interest.
